# TSE Diagnostics: Recent Advances in Immunoassaying Prions

**DOI:** 10.1155/2013/360604

**Published:** 2013-07-18

**Authors:** Anja Lukan, Tanja Vranac, Vladka Čurin Šerbec

**Affiliations:** Department for Production of Diagnostic Reagents and Research, Blood Transfusion Centre of Slovenia, Šlajmerjeva 6, 1000 Ljubljana, Slovenia

## Abstract

Transmissible spongiform encephalopathies (TSEs) or prion diseases are a group of rare fatal neurodegenerative diseases, affecting humans and animals. They are believed to be the consequence of the conversion of the cellular prion protein to its aggregation-prone, **β**-sheet-rich isoform, named prion. Definite diagnosis of TSEs is determined *post mortem*. For this purpose, immunoassays for analyzing brain tissue have been developed. However, the ultimate goal of TSE diagnostics is an *ante mortem* test, which would be sensitive enough to detect prions in body fluids, that is, in blood, cerebrospinal fluid, or urine. Such a test would be of paramount importance also for screening of asymptomatic carriers of the disease with the aim of increasing food, drugs, and blood-derived products safety. In the present paper, we have reviewed recent advances in the development of immunoassays for the detection of prions.

## 1. Introduction

### 1.1. Prions

Prion is by definition a “proteinaceous infectious particle,” responsible for transmissibility of a group of fatal neurodegenerative diseases that affect humans and many other mammals. The so-called protein-only hypothesis, which postulated that the aberrantly folded protein is able to infect and replicate, made prion diseases (at that time quite heretically) distinct from infections caused by microorganisms [1]. 

Prion (PrP^Sc^) has an endogenous cellular counterpart, named prion protein (PrP^C^), which is expressed on the surface of various cell types, most abundantly in the central nervous system. PrP^Sc^ and PrP^C^ share the same amino acid sequence, but differ substantially in the secondary, tertiary, and quaternary structures. PrP^Sc^ is believed to be acting like a mold for converting endogenous PrP^C^ molecules into new prions. However, not only one, but several prion strains have been characterized so far, differing in their structure and biochemical characteristics [2, 3]. Moreover, PrP^C^ as well as PrP^Sc^ can be found in fragments of various lengths [4–6]. Therefore, considering the complex biochemical nature of the target, the difficulty of its detection is obvious.

### 1.2. Transmissible Spongiform Encephalopathies

In humans, the so-called transmissible spongiform encephalopathies (TSEs) or prion diseases have been known to either occur sporadically (sporadic Creutzfeldt-Jakob disease (sCJD)) or can be inherited (familial Creutzfeldt-Jakob disease, fatal familial insomnia, and Gerstmann-Sträussler-Scheinker syndrome). The transmissibility of these diseases was first demonstrated by Gajdusek et al., who successfully transmitted kuru to chimpanzees [7]. However, TSEs came to public attention two decades later when the first case of bovine spongiform encephalopathy (BSE) was reported in United Kingdom, followed by an epidemic outburst of the disease in which more than 180 thousand animals have been diagnosed as BSE positive, and the estimation is that 1–3 million infected animals were slaughtered for human consumption before developing clinical signs [8]. The source of the infection was found to be the prion-infected meat and bone meal, produced from waste parts of sheep and cattle. About ten years after the appearance of BSE, the first cases of new variant CJD (vCJD) have been diagnosed in young patients in UK and later also in some other countries. vCJD was soon connected to the consumption of meat from BSE-infected cattle [9]. Despite the fear, the vCJD cases did not reach the numbers of BSE epidemics (224 cases were described worldwide; http://www.cjd.ed.ac.uk/documents/worldfigs.pdf, 176 from which in UK; http://www.cjd.ed.ac.uk/documents/figs.pdf). However, it was shown that those young and active people, who have been carrying prions for years, before the outbreak of clinical signs, have transmitted them through blood donations. To date, four cases of probable and two cases of possible transmission of vCJD by blood transfusion have been described [10], but as blood or blood products of infected donors was given to many more (http://www.cjd.ed.ac.uk/TMER/summary.htm), the fear exists that more will fall ill. Apart from that, iatrogenic transmission of CJD has been connected to the use of infected surgical instrumentation, to the transplantation of cornea and dura mater grafting (228 reported cases), and to the application of cadaver-derived gonadotropin and human growth hormone (230 reported cases) [11]. 

### 1.3. TSE Diagnostics

Due to the remarkable biochemical diversity among prions on one hand and the disturbing presence of PrP^C^ on the other, as well as due to the absence of specific nucleic acids, TSEs testing has remained one of the biggest challenges of diagnostics until now. 

One way to assess the problem is to search for surrogate markers. For antemortem diagnosis of CJD, different liquor proteins, such as 14-3-3, Tau, phospho-Tau, amyloid-*β* 1–42 and some others (for review, see [12]), have been employed in tests that reach considerably high sensitivity and are often used for CJD diagnostics complementary to neurological signs. However, it is important to stress that the presence of none of them is 100% specific for prion diseases and so far, their use in diagnostics has been limited to the advanced stages of the disease. With the development of new biomarkers and methods that would enable their detection at preclinical stages, liquor diagnostics is expected to take an even more important part in antemortem diagnostics of prion diseases in the near future.

Another way to approach TSE diagnostics is to exploit the physicochemical differences between PrP^C^ and PrP^Sc^. Namely, PrP^Sc^, being richer in beta sheet content, was found to be much more resistant to denaturation and proteolytic degradation than PrP^C^. Ever since, PrP^Sc^ has been detected either by immunohistochemistry (IHC) after special pretreatments of tissue slices, which destroyed relevant PrP^C^ epitopes, or by western blotting of brain homogenates after degradation of PrP^C^ by proteinase K (PK). Many other commercially available diagnostic immunoassays that have been developed still relay on PK digestion of PrP^C^. Contemporary options of discrimination between PrP^C^ and PrP^Sc^ exploit the aggregation-prone nature of PrP^Sc^ molecules in confrontation with to the monomeric PrP^C^.

PrP^Sc^-specific monoclonal antibodies (mAbs) have always represented an ideal approach for prion diagnostics development. However, with the knowledge of various infectious prion strains and fragments, the idea of producing one mAb that would detect them all appears less credible.

In the present paper we have reviewed immunoassays designed to detect pathological form of prion protein as a diagnostic or research tool, discussing their evolution, their advantages, and their weaknesses. Because of the abundance of PrP^Sc^, brain tissue is the most common and reliable diagnostic material. Routine testing of brain tissue is a good way to identify and remove diseased animals from the food chain, and many important advances have been achieved in this area in recent years. Nevertheless, detection of prions at presymptomatic levels of the disease in samples other than brain is the ultimate goal for which researchers still strive.

## 2. Immunoassaying Prions

### 2.1. Detection of Prions in Brain

**Figure 1:**
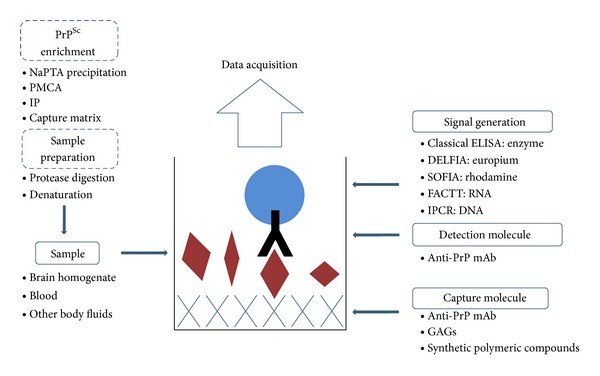
Schematic representation of described PrP^Sc^ immunoassays. Dashed lines indicate optional steps of sample pretreatment.

Several types of ELISA or similar immunoassays have been developed for detection of PrP^Sc^ in brain tissue (Table 1 and Figure 1). PrP^C^ degradation by PK is still the most frequently used sample treatment prior to detection and analysis of PrP^Sc^ and can successfully be transferred from western blot (WB) to ELISA format [13]. ELISA enables simultaneous analysis of larger number of samples than WB, which represents a major advantage. After elimination of PK-sensitive PrP, the remaining resistant forms (PrP^res^) can be detected. PK digestion has in the recent years become somehow controversial. A number of studies have identified PK-sensitive PrP^Sc^ strains, and it is believed that as much as 80% of PrP^Sc^ is PK sensitive [14–19]. Complete PrP^C^ removal and preservation of the whole PrP^Sc^ at the same time is, therefore, hard or in some cases impossible to achieve. The determination of the existence of PK-sensitive PrP^Sc^ strains raised the fear of resurgence of BSE due to the false negative results of routine testing as a consequence of using PK-based tests. Besides, when dealing with tests that rely on enzymes, the adequacy of storage conditions is of considerable importance, as the loss of the enzymatic activity may cause deceptive results. These issues are not to be overlooked since many routine diagnostic methods, especially for BSE, are still based on detection of PrP^res^. Differential resistance of prion strains to PK digestion, which usually poses a problem, can also be exploited for their distinction. Classical scrapie strain, for example, can be distinguished from more sensitive atypical scrapie strain based on the difference in resistance to low and high concentrations of PK [20]. After mild PK digestion both classical and more sensitive atypical strains appear PK resistant. PK in higher concentrations further degrades PrP^Sc^ in atypical strain, destroying relevant epitopes, while epitopes on PrP^Sc^ in classical strain are preserved. The ratio of the signal after mild and harsh digestion is the measurement of sensitivity of certain strain [20].

During the transition from PrP^C^ to PrP^Sc^, and more importantly during the aggregation of PrP^Sc^ molecules, certain epitopes become inaccessible. Upon denaturation of PrP^Sc^, immunoreactivity is greatly enhanced presumably because the structure of the aggregates loosens and buried epitopes become accessible again [21]. Conformation-dependent immunoassay (CDI) exploits this fact for analyzing different prion strains [3]. The method is based on denaturation of different prion strains with rising denaturant concentration gradually revealing hidden PrP^Sc^ epitopes. Denaturation profiles obtained for each strain differ from one another, namely, more stable (and less infectious) strains require higher denaturant concentration for dissociation. The measured optical density (OD) increases significantly for infected material after the denaturation and is, therefore, a measure for the amount of PrP^Sc^ in the individual sample. Based on the difference between OD of denatured and nondenatured samples, infected samples can readily be distinguished from noninfected [3].

Denaturation has been used in numerous studies, in most cases with an important simplification of the original CDI method, although the main principle and the name of the method were retained [22–25]. Instead of measuring the denaturation profile of different prion strains, only one concentration of denaturant, was used for revealing hidden PrP^Sc^ epitopes. Because different prion strains react differently to the same concentration of denaturant some strains, especially less stable, might be overlooked this way. However, according to the authors of these reports, the approach was successfully applied to bovine, ovine, elk, and deer tissues [22, 23]. Additional changes were applied to the first suggested CDI [3]. In all assays sensitivity was increased by introduction of the sandwich immunoassay instead of the direct one. The other common modification was the change of denaturation conditions [22, 24, 25]. It was shown that precipitation step with sodium phosphotungstic acid (NaPTA), that was originally present in the protocol, can be omitted, shortening and simplifying the sample processing [22, 25]. Although the use of denaturation for PrP^Sc^ epitope revealing eliminates the need for PK digestion, it can be applied for more efficient elimination of PrP^C^ and therefore for improved discrimination between TSE-positive and TSE-negative samples [23, 25].

In a different set of assays, denaturation step was employed for differential extraction of PrP [26–28]. Samples were subjected first to low and subsequently to high concentrations of denaturant. PrP^Sc^ aggregates were only soluble when the concentration of denaturant was high enough. Comparison of the two fractions in ELISA (enzyme-linked immunosorbent assay) or DELFIA (dissociation-enhanced lanthanide fluoroimmunoassay) enabled the discrimination between infected and noninfected bovine, murine, and human tissues. 

An important issue of immunoassaying brains is the fact that different parts of brain may vary greatly in the abundance of PrP^Sc^, which was shown for animal and also for human brain [15, 23]. Some parts of infected brain may therefore contain only very low amounts of PrP^Sc^. This issue can to some extent be managed by the knowledge of prion distribution patterns that are present in certain TSEs. Low quality of samples can also be the reason for low amounts of PrP^C^ and PrP^Sc^. In such cases, a low OD is misleading. To avoid misinterpretation of results, normalization of detected PrP^Sc^ against detected PrP^C^ in the same sample can be very useful. A ratio between denatured and nondenatured sample (D/N) can be applied for this purpose [3, 29]. 

In sandwich ELISA capture, mAb is adsorbed to the bottom of the well and detector mAb is used to detect antigen bound to the capture Ab. This format requires two mAbs directed against two different epitopes on one antigen molecule. But in a case of aggregated proteins such as PrP^Sc^, it is reasonable to assume that certain epitopes are represented more than once. This assumption is the basis of the so-called aggregation-specific ELISA (AS-ELISA) that detects only PrP aggregates in brain samples [30]. Using the same mAb for capturing and detecting, it is possible to avoid the detection of PrP^C^, which is usually present as a monomer, and observe only aggregates. 

Ligands other than Abs can be used for the purpose of capturing PrP. Glycosaminoglycans (GAGs) that have been found to bind PrP in the cell [31, 32] can be immobilized onto the solid phase in the ELISA test instead of capturing Ab. Higher affinity of GAGs for PrP^Sc^ in comparison to PrP^C^ enables discrimination between normal and scrapie tissue [33]. A protocol for glycotyping of PrP (which can be non-, mono-, or diglycosylated) was also developed based on the binding of different lectins to specific sugar moieties on PrP [33]. This approach provides yet another advantage, since in comparison to WB in common ELISA the information about glycosylation is lost. Apart from GAGs, other polymeric compounds may bind PrP^Sc^ selectively under defined conditions. This principle was successfully exploited in one of the commercially available BSE tests [34]. 

The above-mentioned methods all rely on frozen tissues that are sometimes not available. As IHC is still the golden standard for definite diagnosis of TSE, much of the tissue taken for analysis is paraffin embedded. Because IHC is not a high-throughput method, protocols for detection of PrP^Sc^ from paraffin-embedded tissue by WB have been developed [35, 36]. They can readily be transferred to ELISA test format [37], enabling the analysis of larger number of samples compared to IHC. In the first step, the tissue is separated from the paraffin by subjecting the tissue sections to boil and freeze cycles. In the second step, the collected tissue is disrupted by sonication. Following tissue disruption, samples are analyzed with the chosen method. 

Sensitivity of an immunoassay depends not only on the sample preparation and treatment, but largely also on the detection system. The simplest and most easily accessible is the ELISA format where detection of PrP is achieved via anti-PrP mAb coupled directly or indirectly to an enzyme which produces visible signal after the addition of the substrate. In more sensitive DELFIA, anti-PrP antibody is labeled with lanthanide chelates, most commonly Europium, that emit stable fluorescent signal. DELFIA was used in a number of studies described in this review [23, 24, 26, 28, 38]. To lower the detection limit even further, a surrounding optical fiber immunoassay (SOFIA) was developed [39]. It is based on sandwich ELISA, but instead of an enzyme conjugate, Rhodamine Red X is coupled to streptavidin. Specially designed hardware that enables maximum light collection and very high sensitivity of the method, at the same time, makes SOFIA less accessible for the widespread use. 

Yet another method that was proved to be more sensitive than WB and IHC is immuno-polymerase chain reaction (IPCR). Original protocol exploits the benefits of both specific antigen recognition in ELISA and exponential amplification of DNA in polymerase chain reaction (PCR) [40]. Antigen is captured as in ELISA followed by the addition of biotinilated DNA instead of en enzyme for obtaining the signal. Bound DNA is amplificated by PCR for enhancing the sensitivity of the protocol [41, 42]. The IPCR was applied to classical ELISA for detection of PrP^res^ in hamster and human brain tissues. Because of the afore mentioned concerns about PK-sensitive strains of prions, a need for PK digestion of the samples is a substantial drawback of the method. To our knowledge, IPCR was never applied to denaturation-based PrP^Sc^ immunoassay.

The reports of the development of PrP^Sc^-specific mAb based immunoassay are very limited. Despite of the use of PrP^Sc^—or aggregate-specific mAb—, these immunoassays are still based on denaturation or PK digestion of samples. The V5B2 mAb, first described by our group in 2004 [43], was later discovered to be specific for a truncated PrP, which ends with the residue Y226 of the human PrP [6]. Although this fragment, named PrP226*, can be present in minute quantities also in normal human brain, it accumulates abundantly in aggregates together with the whole PrP^Sc^ in CJD infected brain [29]. Because it is packed into aggregates and is therefore unavailable for V5B2 mAb, denaturation of samples is necessary for efficient discrimination between infected and noninfected tissues. Nevertheless, greater dissociation compared to the use of non-PrP^Sc^-specific anti-PrP mAb between PrP^Sc^-positive and-negative samples has been achieved in a simple, PK-independent immunoassay. A rationale that a PrP^Sc^-specific mAb-based immunoassay would not need any specific preparation of samples therefore does not seem so plausible anymore, at least for immunoassays using brain samples, where PrP^Sc^ is known to be aggregated. 

### 2.2. Detection of Prions in Blood

All the above-mentioned methods were developed for analysis of human and animal brain tissues, and can thus be applied only for postmortem diagnostics. For an *ante mortem* test, the use of blood and other body fluids needs to be applied (for summary of the blood tests for prions, see Table 2 and Figure 1). As PrP^Sc^ is supposed to be present in extremely low amounts in these samples, immunoassays need to include an additional step of PrP^Sc^ enrichment [44–47]. In an effort to reach high sensitivity, the specificity of the method should not be neglected. Even a small percentage of false positive results in blood donor testing would result in an extremely large number of misinterpreted asymptomatic carriers of the disease. Besides the obvious moral concern, there is also a financial aspect of the issue because all possible carriers should undergo additional diagnostic procedures and should stay under constant medical supervision [46]. 

As stated before, the main problem of detecting prions in blood or plasma is the extremely small quantity of prions and a high background of other proteins and PrP^C^; therefore, extreme sensitivity and specificity is a necessity for a blood test. Apart from that, samples of prion infected blood are rare, limited, and only accessible to few laboratories. To overcome that problem, many test developers make use of spiking brain homogenates or PrP^Sc^ isolated from brain into the blood of healthy persons. This might not be the optimal solution of the problem since pathological PrP, if present in blood, not necessarily possesses the same characteristics as that of brain derived. Nevertheless, such studies are important as they represent an insight into the detection limits we are currently able to reach [38, 46, 47]. Besides the sensitivity issue, the susceptibility to PK digestion also represents a problem because a big portion of PrP^Sc^ in blood may be PK sensitive. Tattum et al. addressed both of these problems in their research [46]. They developed a sandwich ELISA for the detection of PrP^Sc^ in samples of whole human blood spiked with vCJD brain homogenate. The sensitivity was enhanced by immunoprecipitation (IP), reaching the pg level [46]. PK was replaced by a metalloproteinase thermolysin, which was shown to readily digest PrP^C^ into small fragments while leaving PrP^Sc^ intact [48, 49]. Whether the thermolysin is appropriate replacement of PK is a matter of discussion. Only a few studies have addressed this question so far, so it could turn out that certain strains of PrP^Sc^ are sensitive to thermolysin digestion, as was shown for PK. 

Instead of the immunoprecipitation, a precipitation on solid-state capture matrix can be performed [47] taking into account that prions readily bind to stainless steel [50, 51]. The precipitation of PrP^Sc^ from blood on stainless steel particles [47] was more efficient than immunoprecipitation with anti-PrP Ab [46]. PrP^Sc^ was also detected in blood from symptomatic vCJD patients, which is a huge step forward in the antemortem diagnostics of prion diseases [47]. However, tests for screening of blood donors represent a separate issue as their sensitivity should be even higher, as well as they should provide an excellent specificity and a low background. Although more efficient, the use of precipitation on solid-state matrix has a drawback in comparison to immunoprecipitation with anti-PrP Ab. Whereas the selection of Ab enables the specificity of immunoprecipitation, precipitation on solid-state matrix is nonspecific. 

Besides PrP precipitation, another way to approach to the sensitivity issue is *in vitro* amplification of PrP^Sc^. The most widely used method for the* in vitro* amplification of PrP^Sc^ is protein-misfolding cyclic amplification (PMCA) [52]. PMCA exploits the fact that PrP^C^ is converted in the presence of PrP^Sc^. PrP^C^ that serves as a substrate and minute amount of PrP^Sc^ in the sample that serves as a template are incubated together to form new aggregates which are then dissociated by sonication. New PrP^C^ is added and incubation and sonication are repeated. Multiple repetitions of aggregation and sonication cycles enable multiplification of PrP^Sc^ to the level, detectable in WB. The drawback of PMCA is the repetition of many cycles, which is time consuming and increases the possibility of arising of false positive results [53]. 

Two recent reports have shown the use of *in vitro* amplification of PrP^Sc^ in combination with immunoassays more sensitive than WB. Chang et al. described a method that combines the *in vitro* PrP^Sc^ amplification similar to PMCA with AS-ELISA and fluorescent amplification catalyzed by T7 RNA polymerase technique (FACTT), named Am-A-FACCT [44]. In the first step, plasma is mixed with healthy brain homogenate and subjected to amplification. Subsequently, newly formed PrP^Sc^ aggregates are captured by an aggregate-specific mAb in AS-ELISA in combination with FACTT [54] where detection is performed via biotin-conjugated DNA template. The transcription of DNA template into RNA is followed by the addition of the RNA-intercalating dye, and the intensity of the emitted light is measured. Incorporating more steps into the procedure may prolong the duration of an experiment and also increases the possibility of experimental mistake, but the sensitivity can be greatly enhanced. According to Chang et al. [44], Am-A-FACTT can detect PrP^Sc^ aggregates in the blood of scrapie-infected mice and chronic wasting disease-(CWD-) infected mule deer in asymptomatic phase. Combination of limited PMCA, IP, and a very sensitive detection system SOFIA conserves the high sensitivity of the method despite the low number of cycles. This approach enabled the detection of PrP^Sc^ in the blood of scrapie-infected sheep and CWD-infected white-tailed deer in the preclinical phase [45]. 

## 3. Conclusion

The knowledge about prions that has accumulated in the last three decades and the use of routine testing of bovine brain for BSE had great impact on reducing the risk of prion transmission. However, for complete prevention on prion transmission through food, drugs, and blood-derived products, the sensitivity of the methods for prion detection must be greatly improved and designed for analyzing low-content prion material.

The latest advances in PrP^Sc^ immunoassaying set the course of development of testing in different directions, all headed for the same goal—the maximal sensitivity and specificity of the method. 

Accumulating reports on PK-sensitive strains of prions have reflected unfavorably on the use of PK-based diagnostics and therefore in novel prion immunoassays, PK is being avoided.

For routine antemortem testing of potential TSE transmitters, a blood test would be most appropriate. In an effort to develop such a test, different obstacles need to be overcome. Firstly, testing systems, developed for brain tissue, cannot be transferred directly to blood because quantities of PrP^Sc^ in blood are much lower than in brain. Secondly, little is known about biophysical properties of PrP^Sc^ in blood which may differ from PrP^Sc^ in brain. Moreover, samples of infected human blood are limited in number and availability, which is an important drawback. However, a recent study by Edgeworth et al. shows that it is possible to detect prions in the blood of symptomatic vCJD patients [47]. Two other studies on experimentally infected animals demonstrated the detection of blood prions also in the asymptomatic phase of the disease [44, 45], reaching a long-expected milestone. However, both methods are quite complex and therefore do not seem to be applicable to large-scale screening blood tests. The question concerning artificial production of prions by *in vitro* amplification in medical institutions also needs to be taken into consideration. 

A simple, inexpensive, high-throughput, and at the same time highly sensitive blood test for prions does not seem to be available in the near future. A more likely solution seems to be large-scale screening for TSE surrogate markers in combination with an extremely sensitive prion test applied only to the identified risk samples.

## Figures and Tables

**Table 1:** Summary of the methods for detection of PrP^Sc^ in brain tissue.

Reference	PrP source	PK	Denaturation	Antibodies	Detection method	Sensitivity*
[30]	rMoPrP, rOvPrP, rBoPrP, rHuPrP, and mice brain	−	−	C: 11G5 D: 11G5-biotin	Sandwich ELISA	6 ng of aggregated PrP
[13]	BSE bovine brain and scrapie ovine brain	+	−	C: 6H4 D: n.r.	Sandwich ELISA	6 pg rPrP/well 30 pg/mL
[33]	Scrapie sheep brain and tonsils, BSE bovine brain, and scrapie hamster brain	+	−	D: SAF70 Secondary AB conjugated with peroxidase	ELISA	3 ng rBoPrP
[41]	sCJD human brain	+	−	C: 1E5 D: 4F7-biotin Streptavidin-biotin-DNA	IPCR	n.r.
[42]	Scrapie hamster brain	+	−	C: 8b4 or 7A12 D: 3F4-biotin Streptavidin-biotin-DNA	IPCR	1 × 104 PrP^Sc^ molecules/mL or 19 fg/mL
[22]	BSE bovine brain	−	0.1 M GdnSCN	C: 6H4 D: rabbit antiserum C15S Swine anti-rabbit Ab-HRP	Sandwich ELISA	1 ug PrP^Sc^/mL
[26]	BSE bovine brain	−	1 M GdnHCl 6 M GdnHCl	C: FH11 D: 3F4-Eu	DELFIA	36 pg PrP/well
[23]	BSE bovine brain, CWD white-tailed deer, mule deer, and elk brains	+	4 M GdnHCl	C: Fab D18 D: recFab HuM-P-Eu	DELFIA	1 ng rec *β*-MBo2M PrP/mL
[27]	Scrapie mouse and hamster brain	−	8 M GdnHCl 6 M GdnHCl	C: 11G5 D: 7A12, 2F8, 8F9, and 8B4-biotin	Sandwich ELISA	0.05–5 ng rHuPrP
[24]	Scrapie sheep brain	−	6 M GdnHCl	C: FH11 D: 8H4-Eu	DELFIA	200 pg rOvPrP/well
[28]	vCJD human spleen and brain	−	2 M GdnHCl 6 M GdnHCL	C: FH11 D: 3F4-Eu	DELFIA	10 pg rHuPrP/mL
[20]	BSE ovine brain and scrapie ovine brain	+	Heath	C: SAF34 D: Bar224-enzyme	Sandwich ELISA	n.r.
[39]	Scrapie hamster and sheep brain, CWD-infected white-tailed deer brain	−	1% SDS	C: 11F12 D: 5D6-biotin Streptavidin-Rhodamine Red X	SOFIA	10 ag rHaPrP, rMoPrP, rOvPrP, and rDePr
[25]	TME hamster brain	+	3 M GdnSCN	D: 3F4 Goat anti-mouse-HRP	ELISA	n.r.
[37]	Paraffin-embedded scrapie ship, CWD white-tailed deer and TME cattle brains	−	Denaturation buffer (not specified)	HerdChek BSE-Scrapie Ag Test C: polyanionic ligand D: anti-PrP-HRP	ELISA	n.r.
[29]	CJD human brain	−	3 M GdnSCN	C: V5B2 D: EM20	DELFIA	n.r.

*We report the sensitivity as provided by the authors because of the lack of sufficient data for converting the results to the united form.

rHuPrP: recombinant human prion protein, rMoPrP: recombinant mouse prion protein, rOvPrP: recombinant ovine prion protein, rBoPrP: recombinant bovine prion protein, rDePrP: recombinant deer prion protein, rHaPrP: recombinant hamster prion protein.

ICSM is not an acronym but a name of two anti-prion antibodies (ICSM 35, ICSM 18).

**Table 2:** Summary of the methods for detection of PrP^Sc^ in blood.

Reference	PrP source	PK	Denaturation	Antibodies	Detection method	Sensitivity*
[44]	Scrapie mice blood and CWD deer and elk blood	−	−	n.r.	FACCT	n.r.

[38]	CJD human blood	+	−	C: 6H4 D: 3F4-biotin Streptavidin-Eu	DELFIA	50 ul recPrP/mL PK-digested plasma 10 pg recPrP/well

[45]	Scrapie sheep blood and CWD white-tailed deer blood	−	1% SDS	C: 11F12 D: 5D6-biotin	SOFIA	n.r.

[46]	Healthy human blood spiked with vCJD brain	Thermolysin	4 M GdnHCl	C: ICSM 10 D: ICSM 35-biotin	Sandwich ELISA	2.8 pg PrP^Sc^/well 150000-fold dilution (10^5,17^)

[47]	vCJD human blood and healthy human blood spiked with vCJD brain	−	Heat	D: ICSM 18-biotin	ELISA	10^10^-fold dilution of vCJD brain homogenate in whole blood

*We report the sensitivity as provided by the authors because of the lack of sufficient data for converting the results to the united form.

rHuPrP: recombinant human prion protein, rMoPrP: recombinant mouse prion protein, rOvPrP: recombinant ovine prion protein, rBoPrP: recombinant bovine prion protein, rDePrP: recombinant deer prion protein, rHaPrP: recombinant hamster prion protein.

ICSM is not an acronym but a name of two anti-prion antibodies (ICSM 35, ICSM 18).
